# Exosome RNA Sequencing as a Tool in the Search for Cancer Biomarkers

**DOI:** 10.3390/ncrna8060075

**Published:** 2022-11-09

**Authors:** Marina Elkommos-Zakhary, Neeraja Rajesh, Vladimir Beljanski

**Affiliations:** 1 Dr. Kiran C. Patel College of Allopathic Medicine, Nova Southeastern University, Davie, FL 33314, USA; 2 Cell Therapy Institute, Nova Southeastern University, Davie, FL 33314, USA

**Keywords:** exosomes, microRNAs, cancer, biomarkers

## Abstract

Numerous noninvasive methods are currently being used to determine biomarkers for diseases such as cancer. However, these methods are not always precise and reliable. Thus, there is an unmet need for better diagnostic and prognostic biomarkers that will be used to diagnose cancer in early, more treatable stages of the disease. Exosomes are extracellular vesicles of endocytic origin released by the majority of cells. Exosomes contain and transport nucleic acids, proteins, growth factors, and cytokines from their parent cells to surrounding or even distant cells via circulation in biofluids. Exosomes have attracted the interest of researchers, as recent data indicate that exosome content may be indicative of disease stages and may contribute to disease progression via exosome-mediated extracellular communication. Therefore, the contents of these vesicles are being investigated as possible biomarkers for disease diagnosis and prognosis. The functions of exosomes and their contents in disease development are becoming clearer as isolation and analytical methods, such as RNA sequencing, advance. In this review, we discuss current advances and challenges in exosomal content analyses with emphasis on information that can be generated using RNA sequencing. We also discuss how the RNA sequencing of exosomes may be used to discover novel biomarkers for the detection of different stages for various cancers using specific microRNAs that were found to be differentially expressed between healthy controls and cancer-diagnosed subjects.

## 1. Introduction

Endosomes, together with multivesicular bodies, contain membrane-bound intraluminal vesicles which originate from the lumen of the multivesicular body [1,2]. The subset of such vesicles will fuse with lysosomes for destruction, whereas others are released into the extracellular space [1,2]. When these multivesicular vesicles unite with the plasma membrane, this releases exosomes, intraluminal vehicles that are released into the extracellular environment [3]. Originally derived from endosomes, exosomes are extracellular vehicles (EVs) that are generally 50−170 nm in diameter, making them the smallest form of EVs [1]. Exosomes are of broad interest due to their function in cell biology and possible therapeutic and diagnostic uses [3]. Exosomes, once thought of as cellular waste products, are now known to be a unique mechanism of cell communication that participate in a variety of biological processes in both health and disease [3].

Exosomes exert their effects primarily by the transfer of both proteins and RNA to recipient cells, where these molecules modulate recipient cell signaling [1]. While numerous studies have examined the biological effects of exosomal proteins, the role(s) of exosomal RNAs are still being elucidated mostly due to the relatively recent development of highly sensitive RNA sequencing methods [3]. These methods can allow researchers to develop diagnostic techniques that are low cost, noninvasive, and capable of early disease detection and monitoring while patients are undergoing treatment. Therefore, the contents of these exosomes are now being investigated as possible biomarkers for disease diagnosis and prognosis. Using RNA sequencing, researchers have discovered that exosomes contain various RNA species. This review provides insights into how the sequencing of exosomal RNA can help us expand exosome applications, and what information we can gather relevant to exosome isolation methods and exosome roles in healthy and pathological states. The value of exosomes for clinical applications is discussed with an emphasis on their potential for diagnosing and discovering novel biomarkers for cancer. The most recent advancements in RNA sequencing will eventually facilitate the expansion of more sensitive diagnostic applications.

## 2. RNA Sequencing for Assessment of Nucleic Acids

RNA sequencing or transcriptome sequencing (RNA seq) is a technology that uses next-generation sequencing (NGS) to evaluate the quantity and sequences of RNA in a sample [4]. It examines the transcriptome to determine which genes encoded in our DNA are activated or deactivated and to what extent. Early RNA seq techniques relied on Sanger sequencing technology which, while innovative at the time, was both low-throughput and expensive [5]. Only nowadays, with the advent and proliferation of NGS technology, have we been able to fully realize the potential of RNA seq [6,7]. A typical RNA seq workflow includes the following steps: RNA extraction, reverse transcription into complementary DNA (cDNA), ligation, amplification, and sequencing [4]. After obtaining an RNA sample for sequencing, the first stage in the process is transforming the population of RNA to be sequenced into a complementary DNA (cDNA) library [8]. This is accomplished using reverse transcription and permits the RNA to be used in an NGS procedure. The cDNA is then fragmented, and adapters are attached to either end of the fragments. These adapters contain functional features such as the amplification element and the principal sequencing priming site that allow for sequencing to proceed [8]. Following amplification, size selection, clean up, and quality verification, the cDNA library is evaluated by NGS, yielding short sequences that correspond to all or part of the fragment from which it was obtained [4]. The depth to which the library is sequenced differs depending on the purpose of the output data. However, it is crucial to be aware of limitations with library construction methods for RNA seq. High throughput NGS library preparation is made possible for most sample types by the automation of RNA and DNA sample preparation operations [9]. Inherent ligation side products created during library preparation and high sample input requirements have prevented small RNA (sRNA) sequencing from being used more widely [10]. The adapter dimer, a side product, is sized very similarly to the tagged library. Thus, to separate the tagged library from adaptor dimer impurities, most sRNA library preparation methods include a gel purification step. However, the ability to automate library preparation for sRNA is severely limited with gel purification, restricting high-throughput research [11]. The reaction is also dominated by adapter dimer side products at very low sample inputs, which limits this technique’s sensitivity as well [11]. Once the cDNA library has been evaluated, sequencing can be performed using either single-end or paired-end approaches. Single-read sequencing is a less expensive and quicker technology for sequencing cDNA fragments from only one end. Paired-end approaches are more costly since the sequencing occurs from both ends, but they offer advantages in post-sequencing data reconstruction such as investigating the 3′ end region of a fragment and the poly-A tail length simultaneously [4].

Microarrays were previously used to evaluate gene expression prior to the development of RNA seq technique. However, RNA seq is a more flexible and robust technique as it is not confined to known genomic sequences [4]. Since RNA seq does not rely on probes, new RNA sequences can be discovered without reference genes. Thus, RNA seq can help identify new transcripts, alternative splice variants, single nucleotide polymorphisms (SNPs), insertions/deletions, and other changes in RNA sequences [12,13]. RNA seq may also be used to estimate RNA expression levels more precisely than microarrays, which rely on relative quantities rather than absolute numbers. RNA seq offers a window into a cell’s RNA environment throughout distinct physiological or pathological states or stages of development, allowing the assessment of cellular responses based on RNA expression [8]. RNA seq also enables high-throughput NGS by giving both qualitative and quantitative information about the many RNA species present in a sample [4].

## 3. The Role of Exosomes in Biomarker Development

**Figure 1:**
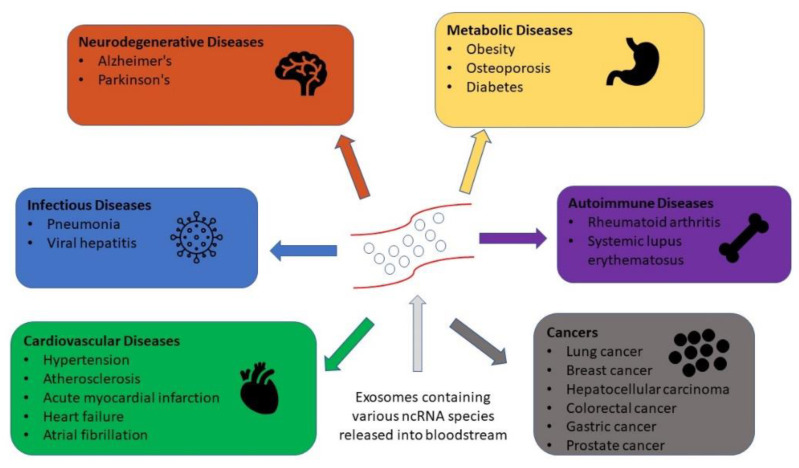
Exosomal ncRNAs in human diseases. Exosomal molecules, including ncRNAs, are dependent on physiological state of the cells they originate from. They are released into the blood stream, allowing them to be utilized as biomarkers of various pathological conditions.

**Figure 2:**
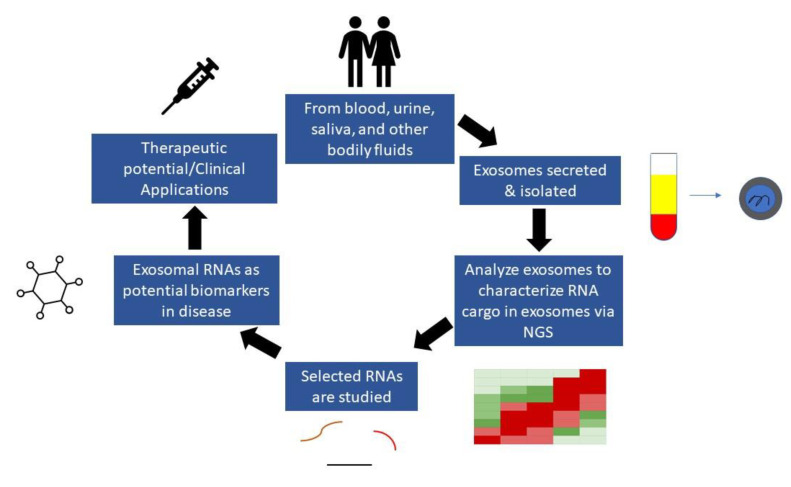
Schematic representation detailing the discovery of biomarkers from exosomes. They are found in and isolated from bodily fluids. The various RNA species from these exosomes are subjected to RNA seq using next-generation sequencing (NGS) to identify potential biomarkers for clinical applications.

Over the last decade, tremendous effort and resources have been devoted to the identification and confirmation of disease and cancer biomarkers [14,15,16]. As a result, numerous studies have been conducted with the goal of precisely identifying and tracking the development of different diseases. For example, carcinoembryonic antigen, carbohydrate antigen 19-9, and carbohydrate antigen 125, often known as CEA, CA19-9, and CA125, respectively, are tumor antigens that can be detected in various cancer types through blood tests [17,18]. These cancers include GI cancers, ovarian cancers, and breast cancer [17,18,19]. However, these cancer biomarkers’ sensitivity and specificity are still inadequate [20,21]. Due to this concern, one study focused on exosomes and reported that exosomal biomarkers possess good sensitivity and specificity in various cancer types [22]. Therefore, the requirement for extremely sensitive and noninvasive diagnostic markers, such as from exosomes, is critical for early disease detection [22]. Thus, the profiling of exosomal RNAs, particularly noncoding RNAs (ncRNA), has been utilized in a number of studies to find novel and extremely promising biomarkers for a variety of illnesses, including neurodegenerative, metabolic, infectious, autoimmune, cardiovascular, and neoplastic diseases (Figure 1) [14]. RNA Seq analysis of isolated exosomal RNA from biological fluids provides the most comprehensive characterization of such RNA and can be used in functional studies or in the discovery of biomarkers that could be used in the clinic for diagnosis, prognosis, or for assessing therapeutic response (Figure 2). As a result, there is an urgent demand in the research and medical fields for rapid and simple ways of isolating exosomes and their subsequent robust and sensitive examination [14].

Due to the lipid bilayer membrane, exosome content is resistant to exogenous proteases and nucleases, making them appealing diagnostic/prognostic tools [3]. Importantly, accumulating evidence indicates differences in exosomal RNA profiles in subjects diagnosed with various conditions compared to those isolated from healthy controls. As a result, exosome-based diagnostic tools for cancer, diabetes, and other disorders are being investigated. Exosomes contain unique RNA and protein cargos that are reflective of the cell of origin and have a wide range of biological functions, including cell-to-cell communication and signaling [23]. Thus, exosomes have enormous promise as biomarkers and might pave the way for the development of less invasive diagnostics and facilitate next-generation therapeutics in the coming years. 

## 4. Isolation of Exosomes from Biological Fluids

Because the field of exosomes is relatively new, there are no standardized methods for their isolation and purification. Various methodologies are now employed for exosome separation and purification; however, there is no agreement on which method is superior [1]. Exosomes have been isolated from human blood plasma [24], serum [25], amniotic fluid [26], saliva [25], and urine [27]. Past studies demonstrated that the final outcome is heavily dependent on the separation process used, since each approach has limits that impact the purity and quantity of the exosomes [28,29,30]. The ideal method of exosome isolation for clinical diagnostics has the following characteristics: low sample contamination, preservation of vesicle integrity, high yield, reproducibility, universality (isolation from all biological fluids), low cost, and high rate of simultaneous isolation from a large number of samples (ideally for no more than one hour) [31]. The accessibility and simplicity of the equipment, as well as process automation, is also crucial. All of the existing conventional exosome isolation procedures are time consuming and labor intensive, have an inconsistent target product yield, and need particular sample preparation [32]. The fundamental issue, however, is the contamination of exosomes with numerous nonexosomal components of biological fluids, particularly when isolated from a complex fluid such as blood. Today, the best alternative for overcoming the problem appears to be a mix of multiple isolation techniques; however, this significantly increases the time required for analysis and its cost, limiting its usage in diagnostic applications [33]. Most methods currently in use are all predicated on vesicle characteristics that are known to be physical (size, shape, density, charge) or chemical (membrane surface composition). Much research has been conducted recently with the goal of comparing various techniques and how they might be used to isolate exosomes from diverse bodily fluids [28,30,34]. These studies have demonstrated that the exosomes isolated from the same biomaterial using various techniques can differ significantly in terms of particle yield and purity as well as in terms of the physical characteristics of nanovesicles (morphology, size), as well as in terms of their biochemical makeup (the level of surface markers, microRNA, and proteins spectrum). Ultracentrifugation, the separation of particle mixtures using a centrifugal force, is the technique most frequently employed to isolate exosomes as it is low cost [1,35]. The technique is based on various rates of sedimentation of particles with varying sizes and densities. Differential centrifugation, rate-zonal gradient centrifugation, and isopycnic gradient centrifugation are the three centrifugation-based techniques [1]. With density gradient ultracentrifugation, more “pure” exosomes can be separated. Density gradient centrifugation, as opposed to differential centrifugation, separates particles in a multicomponent sample that are similar in size (rate-zonal) or density (isopycnic) [1]. Some disadvantages to ultracentrifugation, however, is that it is time consuming with low exosome recovery and the yield can present with nonexosomal impurities [36,37]. In addition to ultracentrifugation, size exclusion chromatography and other solvent-based methods are also frequently used for EV isolation [38]. Size exclusion chromatography allows for EVs to be separated first from smaller particles. However, this method does not provide a high yield of exosomes as well. Thus, the overcompensation of biological fluids is needed for a large yield [39]. Immunoaffinity methods can also be utilized to selectively extract exosomes from complicated biological fluids. Immunoaffinity techniques take advantage of the many proteins, receptors, lipids, and polysaccharides found on the outer surface of exosomes [3]. The fundamental advantage of the immunoaffinity techniques employed for this purpose is that the resultant separated exosomes are of a higher quality and purity. However, the exosomal recovery with this method is also typically poor [40]. As antigen antibody interactions are very specific, this approach may also be used to extract a subset of extracellular vesicles without contaminating them. Overall, future investigations should determine the most beneficial method to isolate exosomes.

## 5. Types of RNAs in Exosomes and Their Biological Functions

The biological function of exosomes depends on the cargo they transport. Due to this, exosomal RNAs, which are typically more diverse than other molecules in exosomes, can provide ample information about the cells they originate from [14]. To this end, exosomal RNA sequencing revealed a mixture of coding and noncoding RNA molecules. It is important to uncover the biological function of these RNA molecules in cells to utilize their diagnostic potential. Among these molecules, there are three main types of RNA species that regulate protein synthesis. First, messenger RNAs (mRNAs) function by carrying codes from DNA, which then may be delivered to recipient cells via exosomes to be translated into proteins [41]. Then, translational RNAs (tRNAs) function by translating mRNA into proteins in ribosomes [42]. Finally, ribosomal RNAs (rRNAs) create the core of ribosomes, which assist mRNA and tRNA to form proteins [42]. Furthermore, there are RNA species involved in gene and RNA regulation, RNA splicing, as well as RNA interference. For example, microRNAs (miRNAs) are delivered via exosomes and function by regulating gene expression post-transcriptionally by binding to mRNAs. It is also important to note that miRNAs have been previously found to be involved with disease progression and various biological processes [43,44]. Piwi-interacting RNAs (piRNAs) specialize in transposon repression in germ cells [45]. Additionally, small nuclear RNA (snRNA) splice introns from pre-mRNA [42]. There are also RNAs that regulate alternative splicing and transcription. These include circular RNAs (circRNAs) and they are able to form a loop through a covalent bond between the 5 and 3 end. [42]. In addition to gene expression, long noncoding RNAs (lncRNAs) can also regulate transcription [42]. Finally, small nucleolar RNA (snoRNA) function by synthesizing ribosomes and regulating rRNA [42]. Nevertheless, small noncoding RNAs have been observed to be the most common type of RNA found in exosomes [14]. Small noncoding RNAs include miRNA, piRNA, snRNA, and snoRNA, while lncRNA and circRNA are long noncoding RNAs [42]. One study reported that exosomes isolated from blood serum and urine samples contain notable amounts of tRNA as well as piRNA, snRNA, and snoRNA [14]. Interestingly, both serum- and urine-derived exosomes contained similar types of tRNA, mRNA, and miRNA profiles. Other studies have indicated that EVs contain significant amounts of miRNA and segments of other RNAs, such as lncRNA, tRNA, and sets of small noncoding RNAs [46,47]. Moreover, a deep-sequencing analysis revealed copious amounts of rRNA with fragments of noncoding RNA from exosomes isolated from urine [48]. Understanding exosomal RNAs’ biological functions will contribute to the evaluation of their potential use as cancer biomarkers.

## 6. Exosomal Secretion from Cancer Cells

**Figure 3:**
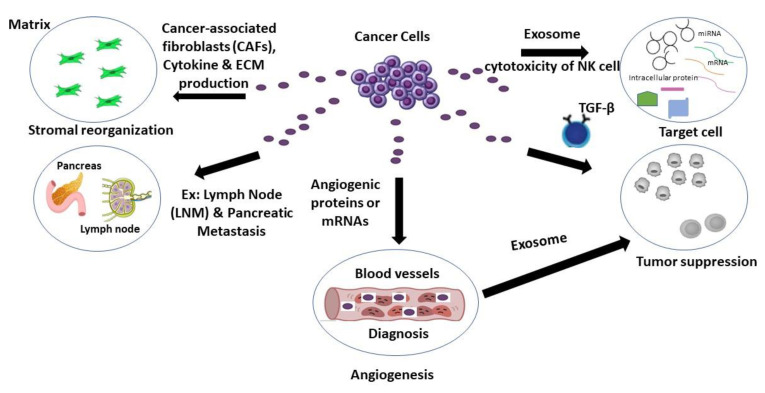
Cancer cells secrete exosomes which can promote metastasis, tumor suppression, cancer-associated fibroblast (CAF) formation, angiogenesis, and the secretion of cytokines. The biomarkers discovered from these exosomes may be useful in detecting various types/stages of cancer.

Studies have shown that exosomes released from cancer cells support metastatic dissemination, facilitate treatment resistance, and contribute to angiogenesis [49]. Additionally, these exosomes can also promote cancer cell motility, invasion, and the secretion of proinflammatory cytokines via decreasing the expression of the genes involved in tumor suppression and extracellular matrix breakdown [49,50,51,52,53]. The exosome miRNAs produced from cancer cells have also been shown to play a prominent role in regulating the activity of stromal cells, namely transforming fibroblasts into cancer-associated fibroblasts (CAFs), which facilitates metastasis for many cancers by reorganizing the extracellular matrix and releasing angiogenic factors [49] (Figure 3). Thus, exosomes carry unique molecular signatures that can serve not only as potential biomarkers in cancer detection, but also allow for the monitoring of disease progression. Below we examine exosome biomarkers in relation to various cancer types. Since exosomal RNA seq has become a standard method in cancer research in recent years and can give significant information regarding a variety of malignancies, this review aimed to concentrate its emphasis on studies that use RNA seq to examine exosome noncoding RNAs in cancer. Furthermore, the cancers chosen were those with the highest potential use of exosomes and various RNA species in diagnostic applications.

### 6.1. Colorectal Cancer

Colorectal cancer (CRC) is regarded as a common gastrointestinal malignancy with an increasing incidence rate [54,55]. One study isolated exosomes from the human CRC cell lines HCT116 and SW620 as well as from the sera of healthy donors. RNA seq revealed that in these exosomes, levels of miR-146a-5p, miR-199-3p, miR-499a-5p, miR-233-3p, and miR-155-5p were the most upregulated compared to the healthy controls. This analysis revealed the upregulation of miR-146a-5p and miR-155-5p; the two miRNAs were found in this study to enhance CAF activation by targeting the JAK2-STAT3/NF-kappa B signaling pathway [49]. Moreover, CAFs cocultured with exosomes enriched with miR-146a-5p and miR-155-5p were also able to promote cancer metastasis. Thus, an RNA seq of exosomes revealed that exosomal miRNA miR-146a-5p and miR-155-5p can be used as potential biomarkers of CRC metastasis [49]. Another study that examined exosomes from CRC cells evaluated the effects of such exosomes on suppressing natural killer (NK) cell cytotoxicity. Upon an RNA sequencing of exosomes, a total of 95 differentially expressed lncRNAs were found. From this, 39 were identified to be downregulated while 56 were upregulated. lncRNA SNHG10 was then identified to be the most enriched in EMT-exos, i.e., exosomes derived from CRC cells undergoing epithelial–mesenchymal transformation, compared with nonEMT-exos. This RNA can prevent the cytotoxicity of NK cells by increasing levels of inhibin subunit β C expression, which is involved in the TGF-β signaling pathway. Thus, these exosomes were able to inhibit NK cell cytotoxicity, suggesting that CRC-derived exosomes can suppress the antitumor activity of NK cells via lncRNA SNHG10 [56]. In a different study, plasma-derived exosomes from both early-stage CRC patients and healthy individuals were subjected to RNA sequencing. This allowed for the identification of 38 upregulated miRNAs as well as 57 downregulated miRNAs. Three miRNAs, let-7b-3p, miR-139-3p, and miR-145-3p, were all found to be highly expressed in early-stage CRC patients compared with the control group. Thus, this study demonstrated the potential of using RNA seq of exosomal RNA to discover biomarkers for diagnosing early-stage CRC [57]. Wang et al. also used RNA seq to profile the miRNAs found in plasma exosomes isolated from 50 CRC patients and 50 healthy individuals. RNA seq revealed eight upregulated miRNAs and 31 downregulated miRNAs. Interestingly, this study found that miR-125a-3p is poorly expressed in exosomes from CRC patients compared with healthy patients. Furthermore, the upregulation of miR-125a-3p is associated with cancer cell apoptosis via p53 activation. Thus, miR-125a-3p is a promising diagnostic marker in CRC. However, further research with larger samples sizes of plasma exosomes is required to confirm these results [58].

### 6.2. Ovarian Cancer

Ovarian cancer is among the most prevalent and deadly malignancies in women. Unfortunately, the majority of instances of this disease are identified in advanced stages, which significantly raises the risk of metastasis and diminishes the chances of patient survival [59]. A recent study utilized RNA sequencing data of exosomes isolated from ovarian cancer cell lines and ascites from ovarian cancer patients containing tumor cells [50]. This study focused on the upregulation of miR-6780b-5p from 25 miRNAs discovered from RNA seq; this microRNA promotes metastasis by stimulating epithelial–mesenchymal transition (EMT), which was validated by further analysis in this study [50]. Such data indicated that the overexpression of miR-6780b-5p facilitated EMT in ovarian cancer cells, while conversely, the downregulation of miR-6780b-5p reduced EMT. These findings show that ascites-containing exosomes transmit miR-6780b-5p to ovarian cancer cells to facilitate EMT, ultimately aiding metastasis. MiR-6780b-5p has also been linked to cytoskeletal regulation, the Notch pathway, and the MAPK pathway, all of which have been shown to be involved in EMT and tumor metastasis [60,61]. Thus, the RNA seq of exosomes led to the identification of potential miRNA that could be used as a biomarker of advanced ovarian cancer and metastasis [50]. Zhang et al. investigated the exosomes isolated from the plasma of ovarian cancer patients as well as from healthy women. RNA seq discovered a total of 65 differentially expressed miRNAs. Specifically, 34 miRNAs were found to be upregulated and 31 miRNAs were downregulated. The top 10 differentially expressed miRNAs (hsa-miR-106a-5p, hsa-miR-185-5p, hsa-miR-99b-5p, hsa-miR-122-5p, hsa-miR-92b-3p, hsa-miR-584-3p, hsa-miR-744-5p, hsa-miR-92a-3p, hsa-miR-93-5p, and hsa-miR-150-5p) were selected for further analysis. From this, it was revealed that the expression levels of hsa-miR-106-5p, hsa-let-7d-5p, and hsa-miR-93-5p were significantly enriched, while hsa-miR-122-5p, hsa-miR-185-5p, and hsa-miR-99b-5p expression levels were significantly downregulated in exosomes from ovarian cancer patients compared with healthy women. Thus, these miRNAs discovered upon the RNA seq of exosomes are promising biomarkers in diagnosing ovarian cancer [62]. In a different study, plasma-derived exosomes were isolated from ovarian cancer patients (n = 34) and healthy individuals (n = 21). Using RNA seq, this study discovered that from seven upregulated and two downregulated miRNA, miR-4732-5p was the most distinguished between ovarian cancer patients and the control group, making this miRNA another promising diagnostic biomarker [63]. Another study revealed that miR-146a and miR-10a, generated from amniotic fluid stem cells (AFSCs) from mice, can protect against chemotherapy-induced premature ovarian failure (POF) in mice. Based on the deep sequencing of AFSC-derived exosomes from mice and exosomes isolated from mouse fibroblast cells (NIH-31T3), 114 differentially regulated miRNAs were discovered. It was found that two microRNAs, miR-146a and miR-10a, were highly expressed in AFSC-derived exosomes compared with those isolated from NIH-3T3. Among these two, miR-10a was shown to have an antiapoptotic impact on granulosa cells, which are required for oocyte and follicle development and survival [64], which are damaged by chemotherapy. As a result, deep sequencing data help uncover an miR-10a that has a potential therapeutic role to help treat chemotherapy-induced POF [65].

### 6.3. Prostate Cancer

The most prominent malignancy of the male reproductive system is prostate cancer, whose prevalence has steadily increased in part due to a failure in diagnosing this condition early when it is treatable [66]. Thus, prostate-tissue-secreted exosomes may provide early diagnostic biomarkers. In a recent study by Zhou et al., the exosomes obtained from prostate cancer patient plasma contained 64 upregulated miRNAs and 30 downregulated miRNAs. Further analysis of the expression profiles revealed high levels of miR-217 and low levels of miR-23b-3p compared with the exosomes from healthy individuals. MiR-217 and miR-23b-3p both regulate cell proliferation and an overexpression of miR-217 was found to stimulate cancer cell proliferation while the opposite occurred with the downregulation of exosomal miR-23b-3p. Therefore, miR-217 and miR-23b-3p were both discovered via RNA seq as potential biomarkers for the progression of prostate cancer [67]. A separate study evaluated levels of miRNAs by comparing the exosomes isolated from urine of both prostate cancer patients and healthy males. An RNA seq analysis revealed a total of 80 miRNAs with five distinguishable downregulated miRNAs in cancer patients compared with healthy males. These miRNAs included miR-196a-5p, miR-34a-5p, miR-143-3p, miR-501-3p, and miR-92a-1-5p. Further analysis of the expression profiles discovered miR-196a-5p and miR-501-3p to be significantly downregulated, so it was concluded that the two miRNAs need to be further evaluated as potential biomarkers [68]. Guo et al. identified a plasma exosomal miRNA as a predictive biomarker for castration-resistant prostate cancer (CRPC). CRPC is a form of prostate cancer that continues to progress despite medical castration [69]. Exosomes were isolated from plasma samples of treatment-naïve prostate cancer patients and CRPC patients, and an RNA seq analysis revealed a total of 185 miRNAs, with five miRNAs (miR-423-3p, miR-99a-5p, miR-320a, miR-200a-3p, and miR-193a-5p) identified as being upregulated in CRPC patient samples compared with treatment-naïve samples. Further analysis in this study demonstrated miR-423-3p as the most distinguished upregulated miRNA. Therefore, miR-423-3p has a potential to be a novel biomarker in identifying CRPC [70]. Kim et al. performed an RNA seq of exosomes isolated from the urine of prostate cancer patients with biochemical recurrence (BCR), which were compared to those without BCR. BCR, an indicator of disease progression, is the process in which the serum prostate-specific antigen level increases [71]. RNA seq revealed 21 differentially expressed miRNAs, among which miR-532-5p was found to be highly enriched in prostate cancer patients with BCR [72]. In summary, RNA seq of exosomal miRNAs yields data that can be utilized to discover novel biomarkers that are useful in both detecting prostate cancer early and distinguishing between prostate cancer types.

### 6.4. Pancreatic Cancer

RNA sequencing of exosomes has also been utilized as a method to detect novel biomarkers of pancreatic cancer (PaC) [73]. For example, exosomes collected from the plasma of patients with PaC were compared to healthy individuals using RNA seq. This analysis uncovered 30 differentially regulated miRNAs, and miR-196a and miR-1246 were selected for further analysis due to the high abundance in plasma exosomes isolated from PaC patients. It was found that the exosomes derived from PaC patient plasma were abundant in miR-196a, which was enriched in subjects diagnosed with adenocarcinoma of the pancreas and miR-1246, which was enriched in subjects diagnosed with intraductal papillary mucinous neoplasms. Thus, the miR-196a and miR-1246 discovered from RNA seq can be used to distinguish between the two subtypes of PaC as well as to diagnose PaC overall [74]. Serum exosomal miRNAs of PaC patients, healthy individuals, and those individuals with benign lesions were also compared in between the cohorts to determine biomarkers for tumor invasion and/or metastasis [75]. This study reported the expression levels of 11 miRNAs. Six miRNAs were upregulated, and five miRNAs were downregulated. MiR-1226-3p was found to be abundant in both the benign PaC cohort and to a lesser extent in healthy individuals, while a decreased expression of miR-1226-3p was found in exosomes isolated from PaC patients. Therefore, it was proposed that miR-1226-3p can be utilized as an indicator for the early detection, development, and metastasis of PaC [75]. Another study analyzed blood serum exosomes isolated from three groups: individuals with intraductal papillary mucosal neoplasms (IPMN), individuals with PaC, and healthy donors [76]. A total of 347 differentially expressed miRNAs were found using RNA seq; among them, miR-191, mir-21, and miR-451a were highly expressed in PaC and IPMN patients’ serum exosomes compared with the healthy control. Thus, RNA seq indicated miR-191, miR-21, and miR-451a as predictive biomarkers in identifying PaC as well as IPMN [76]. Chen et al. performed RNA seq of serum exosomes from PaC patients and healthy individuals (n = 8 for both groups). RNA seq revealed that the expression of exosomal miR-451a was highly enriched in PaC patients compared with benign cancer patients and healthy individuals. Additionally, miR-451a has been previously linked to metastasis in PaC [77,78]. From this finding, it was concluded that miR-451a has potential to be used in the diagnosis of PaC [79].

### 6.5. Breast Cancer

Breast cancer (BC) is the most commonly diagnosed cancer in women. RNA sequencing of exosomes isolated from the plasma of BC patients with recurrent and nonrecurrent cancer reported 20 upregulated and 34 downregulated miRNAs from BC exosomes. These miRNAs were all associated with cancer-related pathways such as the RAS signaling pathway [80], AMPK signaling [81], and mTOR signaling pathways [82]. Interestingly, only a subset of these microRNAs, miR-150-5p, miR-576-3p, and miR-4665-5p were differentially expressed in recurrent patients. Thus, in the future, these three exosomal miRNAs should be evaluated in larger cohorts as biomarkers for recurrent BC [83]. Chen et al. isolated exosomes from patient-derived CAFs and performed RNA seq to determine 31 differentially regulated miRNAs. MiR-500a-5p was found to be highly upregulated, and previous studies determined that this miRNA was also enriched in other cancers [84,85]. Using BC cell lines, miR-500a-5p was found to stimulate BC cell proliferation and metastasis by binding to ubiquitin-specific peptidase 28 [86]. To understand the role of specific CAF subsets in BC drug resistance, Gao et al. performed an RNA seq of exosomes isolated from CD63^+^ CAFs and CD63^+^-depleted CAFs isolated from BC tissues. CD63^+^ CAFs were examined because an initial analysis from the same study revealed that CAFs enriched with the membrane protein CD63^+^ contribute to tamoxifen resistance via an unknown mechanism [87]. RNA seq revealed three abundant miRNAs, miR-22, miR-148a, and miR-152-3p, which were expressed in only CD63^+^ CAF-derived exosomes. Furthermore, miR-22 was selected as the most highly enriched in CD63^+^ CAF-derived exosomes compared with the CD63^+^-depleted CAF-derived exosomes overall. Upon mechanistic examination, it was revealed that exosomal miR-22 functions by downregulating estrogen receptor alpha and PTEN, prompting tamoxifen resistance in breast cancer cells [87]. Additionally, this finding indicates miR-22 as a potential novel biomarker for detecting tamoxifen resistance in BC [87]. Exosomal miRNAs may also serve as indicators for lymph node metastasis (LNM) in BC. Using an RNA seq of exosomes isolated from plasma obtained from BC patients with and without LNM and healthy women, researchers discovered 43 significantly enriched miRNAs. A profile analysis of these miRNAs indicated that miR-363-5p was highly expressed in plasma-derived exosomes from BC patients compared with the healthy control [88]. A decreased expression of this miRNA was also found in plasma-derived exosomes from patients with LNM compared with patients without LNM and healthy women. Furthermore, miR-363-5p appeared to impede metastasis via modulating its downstream target, platelet-derived growth factor subunit B, suggesting that this plasma exosome-derived miRNA can be utilized as a diagnostic biomarker for lymph node metastasis in BC [88].

### 6.6. Gastric Cancer

With over one million new cases identified each year, gastric cancer (GC) is the fifth most frequent and third deadly malignancy in the world [89]. Because early-stage cancer detection is associated with a better prognosis, discovering/developing biomarkers for GC early diagnosis can be critical in enhancing survival rates [90]. Exosomal RNAs have also been studied for their potential as biomarkers in GC. To this end, Tang et al. evaluated exosomal miRNAs using RNA seq in early-stage GC patients’ exosomes isolated from sera, as well as from healthy individuals. RNA seq detected a total of 66 upregulated miRNAs and 13 downregulated miRNAs. Four serum miRNAs, miR-92b-3p, let-7g-5p, miR-146b-5p, and miR-9-5p, were found to be significantly enriched in patients with early-stage GC compared with the healthy control. Based on these findings, RNA seq was used to demonstrate the biomarker potential that the four serum exosomal miRNAs discovered from early-stage GC patients have in the early diagnosis of GC [91]. In a similar study, Wang et al. isolated exosomes from three healthy donors and six GC patients. RNA seq discovered 15 various RNA species, with approximately 28% of miRNA found in abundance and small amounts of rRNA, piRNA, snoRNA, and snRNA. Furthermore, RNA seq detected 11 upregulated miRNAs as well as 24 downregulated miRNAs. Among the downregulation of seven and upregulation of one differentially expressed miRNAs, three downregulated miRNAs, miR-10401-3p, miR-1255b-5p, and miR-6736-5p, were selected for further evaluation. It was then discovered that the expression of miR-10401-3p, miR-1255b-5p, and miR-6736-5p in serum exosomes from GC patients was considerably lower than in serum exosomes from healthy people. Taken together, these data show that exosomal miR-10401-3p, miR-1255b-5p, and miR-6736-5p could be used as diagnostic biomarkers for GC [92]. Zhang et al. also applied small RNA sequencing to uncover differentially regulated miRNAs using exosomes isolated from GC patient plasma as well as from healthy subjects. Furthermore, exosomes were stratified as those originating from lymph node (GCln), ovarian (GCo), and hepatic (GCl) metastases. RNA seq discovered three differentially expressed miRNAs, miR-10b-5p, miR-143-5p, and miR-101-3p, in GCln, GCl, and GCo metastasis, respectively. Further analysis indicated that miR-10b-5p was significantly upregulated in exosomes isolated from GCln patients. MiR-143-5p was significantly upregulated in exosomes isolated from GCl patients, and finally, miR-101-3p was significantly upregulated in exosomes isolated from GCo patients. These miRNAs were not found to be enriched in other types of GC metastasis as well as in the control (healthy) group. Based on these findings, miR-10b-5p, miR-101-3p, and miR-143-5p are potential biomarkers that could be used to distinguish GC patients with GCln, GCo, and GCl metastasis, respectively [93]. Exosomes extracted from GC patient sera and healthy controls were also analyzed using RNA seq in another study which uncovered 306 miRNAs and 161 piRNAs that were differentially expressed. Seven differentially expressed miRNAs and piRNAs each were selected for further analysis due to a high abundancy in the exosomes derived from GC patients. Among these RNAs, miR-1307-3p, piR-018569, piR-004918, and piR-019308 were found to be significantly enriched in exosomes derived from GC patient’s plasma compared with the healthy control. Furthermore, this study also provided evidence that the exosomes isolated from GC patients with metastasis compared with GC patients without metastasis were enriched in piR-004918 and piR-019308. Thus, the RNA seq of exosomes derived from GC sera uncovered four small RNAs as promising biomarkers in detecting GC as well as GC metastasis [94].

## 7. Exosome, miRNA and RNA Sequencing Use in Future Studies

Because exosomes are found diluted in biological fluids, it is necessary to further simplify their isolation and purification procedures in order to isolate them in larger quantities. This is because current methods are not cost effective and are time consuming. Additionally, exosomal RNA extraction also holds some issues due to a lack of optimized strategies when using limited sample quantities [95]. Furthermore, the biological roles and mechanisms of exosomal RNAs, in vivo, need to be studied more toughly [96]. The function of exosomal miRNA at different stages of cancer must also be investigated, as it may have different functions at different stages of the disease. For example, one study compared miRNA expression levels before and after resection in colorectal cancer patients and discovered that the levels of oncogenic miRNAs had decreased after resection [97]. Thus, future studies should also explore if the exosomes isolated from subjects diagnosed with other cancer types before and after resection also have different levels of exosomal miRNA postresection. This would assist researchers in recognizing which miRNAs function greater as biomarker(s) in cancer patients who have had tumors removed. Early-stage cancers also present a greater challenge to the development of exosomal RNA biomarkers as their expression levels are low at this stage [98]. Additionally, most of the miRNAs discussed in this review are cancer-type specific. However, miR-146, miR-150-5p, miR-196a, miR-143a, and miR-92b-3p were discovered in various types of cancer in this review as well. Thus, it might be of interest for additional studies to explore miRNAs that are not cancer-specific to provide a single miRNA biomarker for a variety of cancer patients. For example, Ferracin et al. demonstrated upon RNA seq that from nine differentially expressed miRNAs, miR-21-5p was highly enriched in all exosomes and other microvesicles isolated from the sera of patients with lung, breast, colorectal, and skin cancer compared with the healthy subjects [99]. However, while discovering a single miRNA biomarker to detect multiple cancer types may be beneficial, it is also important that future studies include an additional analysis of specificity and sensitivity. This would help further validate the promise of these potential diagnostic tools. There is also a lack of investigation on other RNA species besides miRNA as prospective biomarkers for diagnosis and prognosis [100]. With RNA seq, future studies will also need to be conducted regarding reproducibility. It has been revealed that it is difficult for studies to replicate RNA seq studies due to lack of standardized methods [101]. With further investigation, this issue can be resolved to provide high-quality reproducibility for studies using different types of RNA molecules as cancer biomarkers. Both current and future studies are also examining the potential use of exosomal RNAs as cancer therapeutics in preclinical and clinical studies. For example, a preclinical study utilized exosomal RNA to focus on treating pancreatic cancer, specifically the KrasG12D mutation, in multiple mouse models. This was performed using exosomes loaded with small interfering RNA (siRNA), which were derived from mesenchymal stromal cells. This study revealed that this treatment suppressed pancreatic cancer in multiple mouse models, indicating a promising therapeutic approach to target KrasG12D with exosomal RNA [102]. Similar studies will allow further development in exosome-associated therapeutics in the field of cancer diagnosis and prognosis, as well as other various diseases.

## 8. Conclusions

We highlight here the RNA sequencing of exosomes as a discovery tool for establishing biomarkers of various cancer types. While not every cancer type has been addressed, we argue that exosomal RNA has diagnostic power to assist with detection at various stages of the disease. These findings can not only assist future studies but can also serve in cancer prediction, diagnosis, and a therapy follow up in precision and personalized medicine for patients. However, the use of exosomes is currently limited for a variety of reasons, including a lack of a standardized separation and characterization approach and the difficulty to distinguish between exosomes released by normal and malignant cells [103]. As a result, resolving these constraints in order to embrace the unique potential of exosomes to capture the dynamic complexity of cancer should be a high priority.
